# Permanent modifications in silica produced by ion-induced high electronic excitation: experiments and atomistic simulations

**DOI:** 10.1038/s41598-017-11182-4

**Published:** 2017-09-06

**Authors:** Antonio Rivera, José Olivares, Alejandro Prada, Miguel L. Crespillo, María J. Caturla, Eduardo M. Bringa, José M. Perlado, Ovidio Peña-Rodríguez

**Affiliations:** 1 Instituto de Fusión Nuclear, Universidad Politécnica de Madrid, José Gutiérrez Abascal 2, Madrid, E-28006 Spain; 2 Centro de Microanálisis de Materiales, Universidad Autónoma de Madrid, Madrid, E-28049 Spain; 3 Instituto de Óptica “Daza de Valdés” (CSIC), Serrano 121, Madrid, E-28006 Spain; 4 Departamento de Física Aplicada, Facultad de Ciencias, Fase II, Universidad de Alicante, Alicante, E-03690 Alicante, Spain; 5 CONICET and Facultad de Ciencias Exactas y Naturales, Universidad Nacional de Cuyo, Mendoza, 5500 Argentina

## Abstract

The irradiation of silica with ions of specific energy larger than ~0.1 MeV/u produces very high electronic excitations that induce permanent changes in the physical, chemical and structural properties and give rise to defects (colour centres), responsible for the loss of sample transparency at specific bands. This type of irradiation leads to the generation of nanometer-sized tracks around the ion trajectory. *In situ* optical reflection measurements during systematic irradiation of silica samples allowed us to monitor the irradiation-induced compaction, whereas *ex situ* optical absorption measurements provide information on colour centre generation. In order to analyse the results, we have developed and validated an atomistic model able to quantitatively explain the experimental results. Thus, we are able to provide a consistent explanation for the size of the nanotracks, the velocity and thresholding effects for track formation, as well as, the colour centre yield per ion and the colour centre saturation density. In this work we will discuss the different processes involved in the permanent modification of silica: collective atomic motion, bond breaking, pressure-driven atom rearrangement and ultra-fast cooling. Despite the sudden lattice energy rise is the triggering and dominant step, all these processes are important for the final atomic configuration.

## Introduction

High electronic excitation produces a myriad of processes in non-metallic materials: ionization, carrier scattering, carrier diffusion, exciton formation, Auger recombination, non-radiative exciton decay, which are followed by thermal processes such as ablation, heat diffusion and atomic rearrangement^1^. A full consistent description of the underlying physical processes is still missing. Efforts to follow the electronic evolution and its subsequent coupling to the atomic lattice are underway, mainly within the framework of intense fs-laser irradiation, through elaborated quantum kinetic models, semi-classical Monte Carlo approaches and hydrodynamic codes^2–4^. In the case of ion irradiation in the electronic stopping regime (with specific energies, that is, the ratio of ion energy to ion mass, *E*_s_ > 0.1 MeV/u), permanent modification generally occurs for electronic stopping powers above a threshold (S_e_ > S_th_), affecting a region (track) of nanometric dimensions around the ion trajectory^5–9^. The physical processes responsible for permanent damage in this case strongly differ from those in the nuclear stopping regime (*E*_s_ ~ keV/u), dominated by elastic collision mechanisms, which are well established in the literature^10–13^. A detailed description of ion irradiation in the electronic stopping regime presents additional complications with respect to the case of fs-laser irradiation. This is mainly because of the generation of ballistic electrons (δ-electrons) that move away from the ion trajectory producing further ionization (secondary electrons). The ionization problem requires considering the thermalization and 3D transport of electrons and holes under an intense local field generated by the induced charge densities. Several phenomenological descriptions based on different (often contradictory) concepts have been proposed to explain track formation^9, 14–20^. Alternatively, atomistic models have been used to describe the lattice evolution upon irradiation^21–31^. The atomistic models have gained popularity in recent years thanks to the development of new reliable interatomic potentials for many materials, widely accessible codes and the ever-growing computational power. Nowadays, atomistic models are able to accurately follow the thermal and structural evolution of the system after irradiation. However, the atomistic models cannot deal with the details of electronic excitation, just with the subsequent atomic evolution. Several assumptions and approaches must be made in order to convert the energy deposited in the electronic system into lattice energy. Therefore, it is of paramount importance to consider a careful validation of the results obtained with atomistic models with the aid of experimental data, prior to draw general conclusions.

In this paper we concentrate on the case of silica irradiated with ions in the electronic stopping regime. Unfortunately, the experimental results reported in the literature^32–37^ are not as systematic as in the case of crystalline materials, due to the additional difficulty to characterize nm-sized ion tracks in amorphous materials. Thus, common techniques for crystal studies, such as e.g., Rutherford backscattering spectrometry in channelling configuration (RBS-C) or transmission electron microscopy (TEM) become inappropriate for amorphous silica. Alternatively, other methods have been employed. For instance, Fourier transform infrared spectrometry (FTIR) measurements^33^ were used to study the track size, small angle X-ray scattering (SAXS) measurements^32^ to study the track structure and optical absorption measurements^36^ to determine colour centre production.

We carried out systematic series of Br and F ion irradiation (<0.1–0.5 MeV/u) with the same type of samples. The samples were studied by *in situ* optical reflection techniques^38^ to follow compaction as a function of fluence and by *ex situ* optical absorption techniques^39^ to follow colour centre generation. In particular, regarding colour centre formation, to the best of our knowledge, systematic studies with different ion energies are very scarce with a few exceptions, for instance, those reported by Ma *et al*.^36^. The analysis of different effects produced in the same conditions turns out to be a very powerful tool to understand the physical mechanisms responsible for the observed permanent modifications in silica.

The experimental results provide valuable information on damage generation by means of track formation and accumulation. The main features extracted from these observations are: (i) there exists a clear velocity effect ^40–44^, i.e., for a given stopping power, ions with low specific energy produce permanent damage more efficiently (e.g., larger tracks) than ions with high specific energy; consequently, (ii) an associated thresholding effect appears, i.e., the higher the ion specific energy, the higher the threshold (*S*_th_) for permanent damage production; (iii) in general, a complex track structure ^32^ is observed, with a central low density region (*core*) surrounded by a high density region (*halo*), with the overall track density higher than that of the virgin material^37, 38^; (iv) this leads to silica compaction ^37, 45, 46^; (v) the resulting tracks contain irradiation-induced defects due to electronic excitation, some of them optically active (colour centres)^36, 39^, which can be easily detected by optical absorption techniques; (vi) track accumulation leads to a density increase in the irradiated region until a compacted continuous layer ^38^ with a maximum colour centre density is formed; (vii) further irradiation results in plastic deformation ^37^ partially restoring the original density (out of the scope of this work).

In addition to the experimental data, we describe an atomistic model based on large-scale molecular dynamics (MD) to explain the results. The model uses a simple method^21–28^ to account for the energy transferred from the electronic system to the lattice, easily applicable to a large variety of ion energies and materials. The subsequent evolution is quantitatively described by MD. Permanent effects appear in a nanometer-sized track associated to the ion passage with density and defect concentration considerably different to those of the virgin material. New systematic experimental irradiation series with specific energies between 1 and 15 MeV/u would be useful to validate our conclusions, but our current experimental results combined with those from the literature turn out to be appropriate for a quantitative validation of the model. Indeed, the model consistently accounts for very different observations such as the velocity and thresholding effects, complex track formation and colour centre generation. This enables its use to draw conclusions about the physical processes involved in damage generation. We will show that both, compaction and colour centre generation, are a consequence of the sudden lattice energy rise and the subsequent evolution during ultra-fast cooling down. Compaction is triggered by the initial collective atomic motion and colour centre generation by bond breaking. Understanding the intrinsic mechanisms of damage in silica is of technological relevance due to the importance of silica as the material of choice in many applications subject to irradiation, either unavoidably due to its application function^47–49^ or intentionally to tailor its properties^50^. In this context, the results reported in this paper constitute a significant contribution. Similar approaches can be applied to the study of other technological materials.

## Results

### Experimental observations

In order to study the effects produced in silica by ion irradiation in the electronic stopping regime, we carried out systematic *in situ* optical reflection^38^ and *ex situ* optical absorption measurements^39^ (reanalysed here) during Br and F irradiations according to the details given in Table 1. The combination of both *in situ* optical techniques constitutes a powerful tool to study the ion irradiation effects, as shown next.

**Table 1:** Parameters obtained from the experimental F and Br ion irradiation-induced dielectric constant variation fitted to Equation (1).

Ion and energy (MeV)	Specific energy *E*_s_ (MeV/u)	Surface electronic stopping power, *S*_*e*_ (keV/nm)	Track radius, *R* (nm)	Track dielectric constant variation, Δ*ε*_max_	Track density variation, Δ*ρ* (nm^−3^)	Normalized track density variation, Δρ/*ρ*_0_
F 5	0.25	2.1	1.05	0.0537	2.26	0.034
Br 5	0.06	2.1	1.76	0.0452	1.91	0.029
Br 10	0.13	3.4	2.07	0.0473	1.99	0.030
Br 15	0.19	4.7	2.66	0.0451	1.90	0.029
Br 25	0.31	5.8	3.51	0.0551	2.32	0.035
Br 40	0.50	7.2	3.77	0.0526	2.22	0.034

In addition, the resulting average track density obtained from Equation (2) is shown. The surface electronic stopping power was calculated with SRIM^13^.

**Figure 1:**
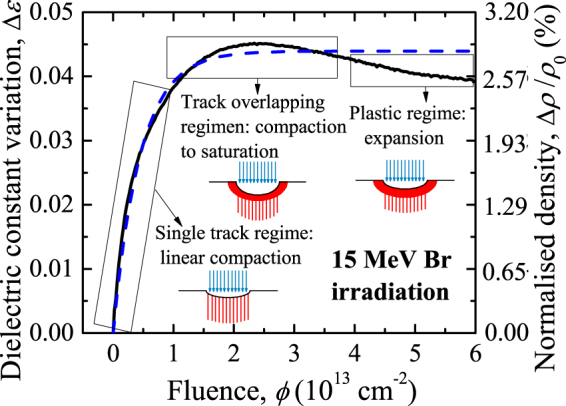
Dielectric constant variation as a function of fluence for 15-MeV Br irradiation. This variation is transformed into density variation by means of Equation (2) and indicated by the right axis. The dashed line is the fit of the experimental data to Equation (1). The insets illustrate schematically the three regimes in track formation and accumulation.

*In situ* optical reflection measurements provide information on the effective dielectric constant variation (due to track accumulation) in the near surface region during ion irradiation. The dielectric constant is obtained from the reflectance by means of the Fresnel equations^38^. An example of *in situ* reflectance measurements with 15 MeV Br ions is given in Fig. 1. Similar curves are obtained in all our irradiations. Three different regimes can be clearly distinguished: (i) linear dependence at low fluences (*single track regime*); (ii) sublinear behaviour up to a maximum at intermediate fluences (*overlapping track regime*); (iii) gentle decrease to a saturation value at high fluences due to plastic deformation (*plastic regime* ^37^). The thermomechanical response of silica in the *plastic regime* is out of the scope of this paper; however, it is interesting to note how the *in situ* optical measurements are well suited to follow this effect.

In the *single track regime*, the near surface region contains both virgin material and isolated ion tracks. Consequently, the effective dielectric constant variation, Δ*ε*, in this regime increases linearly with the irradiation fluence, *ϕ*. The derivative at the origin provides the effective dielectric constant variation per track. As the fluence increases the dielectric constant variation deviates from the linear regime because tracks start to overlap (*overlapping track regime*). At sufficiently high fluence, corresponding to a fully modified surface, a saturation maximum appears. In these conditions a continuous layer with modified properties appears in the near surface region. This layer grows deeper for increasing fluences until a several-μm-thick uniform layer is formed^38^. Typically, the interfaces between the modified layer and the substrate are very abrupt when irradiating with ions in the electronic stopping regime.

The dielectric constant variation evolves following a Poisson-like kinetics in the *overlapping track regime*. This indicates that ions reaching an already modified region do not produce, as an initial approximation, any new effect. This is obviously not the case in the *plastic regime*. The Poisson-like kinetics can be written as

1 $${\rm{\Delta }}\varepsilon ={\rm{\Delta }}{\varepsilon }_{{\rm{\max }}}[1-\exp (-\sigma \varphi )]\,{\rm{with}}\,\sigma =\pi {R}^{2},$$

where *σ* represents the track cross section, *R* the track radius and Δ*ε*_max_ = *ε*_max_ − *ε*_virgin_ the dielectric constant variation in the near surface region when a continuous layer is formed. Thus, *ε*_max_ corresponds to the average dielectric constant of the surface region in the *overlapping track regime*, being *ε*_virgin_ the dielectric constant of the virgin material. The average dielectric constant of a track coincides with *ε*_max_ and it is related to its average density by means of a modified Lorentz-Lorenz model^51^:

2 $$\varepsilon =\frac{2\rho +8.0324}{8.0324-\rho },$$

where *ρ* represents the average density in g/cm^3^. The track parameters can be obtained by fitting the F and Br experimental curves to (1), as shown in the example of Fig. 1. The relation between dielectric constant variation and density variation is shown in the figure with the right axis and evidences the compaction processes. An advantage of *in situ* optical reflection measurements is that they provide a direct way to monitor compaction by means of track generation and accumulation.

**Figure 2:**
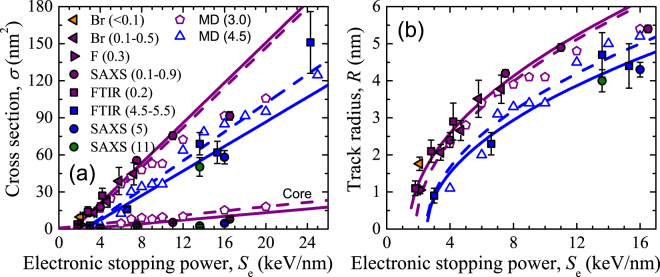
(Solid symbols). Track cross sections (**a**) and the corresponding track radii (**b**) as a function of the electronic stopping power for irradiations with ions of different specific ion energies given in parentheses in MeV/u and indicated with different colours. In addition to our F and Br irradiation series studied by *in situ* optical absorption measurements, we include experimental data from the literature obtained by SAXS^32^ and FTIR^33–35^. (Open symbols). MD simulations carried out with the hot cylinder radius given in parentheses in nm. The SAXS data corresponding to the core region reported by Kluth *et al*.^32^ is included in (**a**) and compared with the MD results. The continuous lines are linear fits to the linear part of the experimental cross sections, whereas the dashed lines are the linear fits to the MD cross sections. The curves in (**b**) are the fits in (**a**) for cross section converted to radius as follows, *R* = (*σ/π*)^½^. The MD radii are accompanied by an error of ±0.5 nm.

The parameters of the tracks generated in our Br and F irradiation series are listed in Table 1. Track radii increase with increasing stopping power, whereas the average track density increases to ~3% in all cases, in agreement with published data^37, 46^, regardless of the stopping power. Figure 2(a) shows the track cross sections at the silica surface as a function of the stopping power for F and Br irradiations in addition to the few systematic data sets reported in the literature^32–35^ and MD results, which will be described in the next section. Figure 2(b) contains the corresponding track radii. Note that different techniques provide systematically different cross sections (radii), nevertheless, the velocity effect^40–44^ becomes evident: the lower the ion specific energy (for a given stopping power) the larger the cross section (radius). In other words, low specific energies are more efficient for track formation than high specific energies. A clear indication of the velocity effect can be observed with the radii obtained by *in situ* reflectance for 2-keV/nm Br and F ions, see Fig. 2(b). These experiments were carried out in the same conditions, therefore, the resulting different radii are not related to any experimental subtlety but to a real physical effect. Br ions (lower specific energy, due to their higher mass) give rise to a larger track radius than F ions (higher specific energy). The cross section depends linearly on stopping power up to rather high values. The linear fit to the data results in different slopes for the series obtained at *E*_s_ < 1 MeV/u and ~5 MeV/u (purple and blue, respectively), a clear consequence of the velocity effect. The velocity effect leads to the existence of a threshold value above which permanent modifications occur. The thresholds can be obtained by means of a linear extrapolation to *σ* = 0 providing a value *S*_th_ = 1.4 keV/nm for *E*_s_ < 1 MeV/u, considerably smaller than the value *S*_th_ = 2.5 keV/nm obtained for *E*_s_ ~ 5 MeV/u.

Kluth *et al*.^32^ experimentally observed that the tracks present a complex structure with a low density core and a high density halo. Figure 2(a) includes the cross sections of some track cores (*σ*_c_). The series obtained with specific energies < 1 MeV/u contains four points. By means of them a threshold for core formation can be obtained. It turns out to be 2.7 keV/nm, much higher than the threshold for track formation obtained above. Unfortunately, no systematic SAXS series have been reported for higher specific energies. Therefore, for 5 and 11 MeV/u (blue and green, respectively) we cannot obtain the threshold values for core formation, however, the data points indicate that they are considerably higher than for *E*_s_ < 1 MeV/u, as expected.

**Figure 3:**
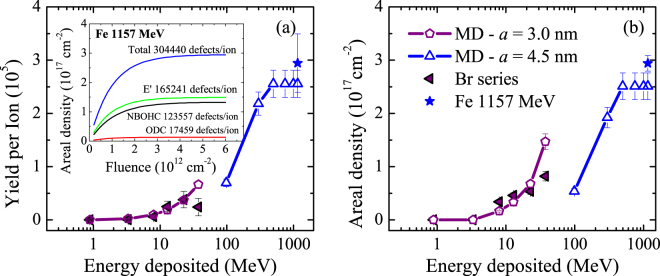
Colour centre yield per ion (**a**) and saturation areal density (**b**) as a function of the ion energy deposited in the electronic system. The solid symbols correspond to our Br irradiation series and the 1157-MeV Fe irradiation experiments reported in the literature^36^. The open symbols correspond to MD simulations carried out with a hot cylinder radius of 3.0 or 4.5 nm to simulate the Br and Fe irradiation experiments, respectively.

Absorption spectra obtained with *ex situ* optical absorption measurements^39^ present different bands, which are associated to different types of colour centres. According to the literature^52–55^, we can assign the most relevant absorption peaks to E’-centres, non-bridging oxygen hole centres (NBOHC) and oxygen deficient centres (ODC). Table 2 summarises the relevant parameters for colour centre analysis. Thus, with absorption measurements, one can extract the areal densities of the different colour centres in the irradiated region (a few-μm-deep in our Br irradiation series). Unfortunately, there are very few systematic examples in the literature on defect generation by ions in the electronic stopping regime. For comparison with our experimental results, we include in Fig. 3 the experimental data obtained by Ma *et al*.^36^ on 1157-MeV-Fe irradiated silica. Typically, the colour centre areal density increases with fluence in a Poisson-like fashion (example in the inset of Fig. 3), similar to that described by Equation (1) for dielectric constant variation. However, in this case, we cannot obtain the track cross section at the surface because the measurements provide information about the whole track, including the nuclear stopping region. The derivative at the origin provides the colour centre yield per incoming ion. In order to obtain the total colour centre yield for every Br irradiation experiment, we added up the E’, NBOHC and ODC yields and subtracted an observed constant contribution in all cases, which is attributed to elastic collisions in the nuclear stopping regime (~5000 defects/ion). We assumed the same background in the Fe data because the number of defects predicted by SRIM^13^ is about the same as for Br irradiation. The total colour centre yield is plotted in Fig. 3(a) as a function of Br and Fe ion energy deposited into the electronic system. The yield increases with increasing ion energy. As observed in the inset, at sufficiently high fluence the colour centre areal density reaches a maximum and saturates. Figure 3(b) contains the total colour centre saturation densities, which depend on ion energy in the same way as the corresponding colour centre yields. The Poisson-like behaviour of the colour centre production indicates that ion irradiation of an already irradiated region does not produce new defects in a first approach. This is the reason for colour centre saturation at a sufficiently high fluence. Thus, the saturation density must be very similar to the single track average colour centre density and explains why the saturation densities increase with increasing ion energies in a similar fashion as the colour centre yields. Further discussion based on MD results appears in the next sections.

**Table 2:** Parameters used for the Gaussian deconvolution of the optical absorption spectra to obtain the contribution of different colour centres^52–55^.

	NBOHC	ODC-II	E’	NBOHC	Extra band	ODC-I
Position (eV)	4.8	5.0	5.8	6.8	7.3	7.6
FWHM (eV)	1.0–1.2	0.35–0.4	0.8–0.9	1.8	0.6	0.5
Oscillator strength	0.05	0.15	0.15	0.05	—	0.4

## Atomistic Simulations

A calculation of the exact energy deposition by an incoming ion of high electronic stopping power requires a 3D quantum mechanical model able to account for the time-dependent energy transfer from the excited electrons to the lattice, which is out of the scope of this paper. However, for the scenario in our experiments, the energy deposited by an incoming ion into the electronic system is rapidly transferred to the lattice and subsequently diffuses away to the surrounding massive cold material, typically resulting in Gaussian-like temperature profiles around the ion trajectory. In our MD simulations, we set the initial total kinetic energy of the atoms in a hot cylinder of radius *a*, in the range 3–5 nm, equal to the electronic stopping power (*S*_e_) of the ion, as previously done in many studies^21–27^. The parameter *a* determines the initial energy density and it is naturally linked to the velocity effect, as we discuss in the next section. Note that at the timescales of the electron-lattice coupling, energy loss channels, such as radiative recombination (luminescence) are of little importance. Therefore, it is assumed that the experimental *S*_e_ is equal to the simulated *S*_e_. This simple energy deposition approach turns out easy to implement and yet very predictive, as we demonstrate in this paper. In a recent study^56^ we used the same methodology to implement MD simulations that successfully explain ion irradiation effects on silver nanoparticles embedded in silica. Detailed information on MD is given in Methods. More sophisticated schemes require input parameters, which usually are not available. We decided to use this simple proven approach based on only one free parameter (the cylinder radius) and treat it as a calibration parameter. The simplicity of the method is beneficial both for the implementation and to provide a physical interpretation to the cylinder radius (next section). Obviously, when the fine details of energy deposition play a role on the studied effect (e.g., studies of ultra-fast transient effects) this method is not applicable, but in our case, these fine details do not determine the final network evolution after cooling and thus, the method becomes very useful, as shown in this paper.

**Figure 4:**
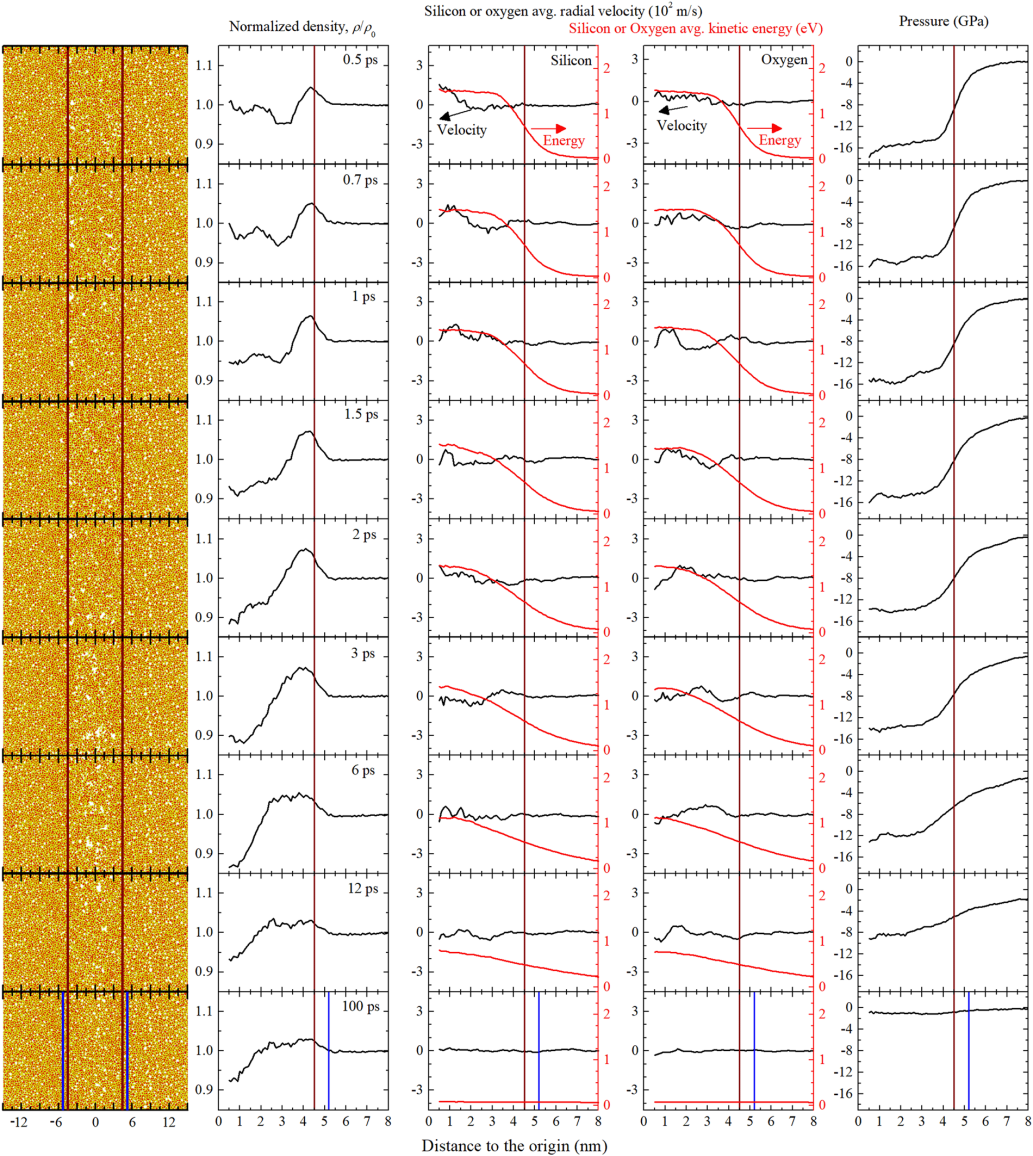
From top to down, evolution of the silica system at indicated times after energy deposition in a hot cylinder of radius *a* = 4.5 nm (represented by vertical lines). Left column. Silicon and oxygen atoms in the simulation box used for MD simulations. An ion with an electronic stopping power *S*_e_ = 16 keV/nm is supposed to travel across the box along the vertical axis. Only atoms in a central 1-nm-thick slice are shown to visualize the initial atom depletion in the centre and the subsequent partial reconstruction. The meaning of the other columns is given in the titles. The last row (100 ps) contains additional vertical lines to indicate the resulting permanent ion effect (permanent track). Every point in the figures is obtained by considering all the atoms at the indicated radial distance from the track centre ±0.5 nm.

An example of a nanotrack simulation in silica is given in Fig. 4 for ions with *S*_e_ = 16 keV/nm. The energy deposition takes place in an initial hot cylinder of 4.5 nm resulting in a sudden energy increase around the ion trajectory. The kinetic and potential energies are already equilibrated at 0.5 ps. At this time Gaussian-like temperature (kinetic energy) profiles appear. In addition, a collective (subsonic) motion of atoms in the radial direction leads to an effective mass transport out of the hot cylinder region. As a result, the density in the hot cylinder drops dramatically. Meanwhile, displaced atoms accumulate outside the initial hot cylinder, where they meet cold atoms producing a sharp density rise, which extends considerably beyond the hot cylinder radius. The sudden density variation leads to the appearance of a large compressive pressure (−16 GPa) in the hot cylinder region. Net mass transport out of the hot cylinder continues as time elapses, due to the huge thermal gradient and the fact that hot atoms interacting with surrounding cold atoms swiftly thermalize outside the hot cylinder. At 1.5 ps, the normalized density drops down to ~0.9 in the central region and reaches a peak of 1.072. From 1.5 to 12 ps, while the cooling process swiftly takes place, the density profile becomes smoother. This is a sign of atom rearrangement driven by the still very hot track and the large pressure and results in a rather flat density variation profile with a maximum of ~1.03 covering most of the track volume. For times >12 ps, mass transport is strongly suppressed because temperature is already low. However, further diffusion and local rearrangement affects the final defect distribution and contributes to full pressure relaxation. At 100 ps, the whole sample is “cold” and the situation prior to the ion passage cannot be restored. A track is observed with higher density than the virgin material and a complex structure constituted by a markedly low density region in the centre (core) and a high density region (halo) around it. Long-term diffusive rearrangement can play a role in laboratory timescales resulting in further relaxation. Obviously, such effects are not accounted for in our 100-ps scale MD simulations.

The recovery effect is very evident in the first column of Fig. 4, which represents a 1-nm-thick slice of atoms centred along the ion trajectory. Porosity (low density) develops initially in the centre of the hot cylinder, reaching a maximum at ~6 ps. From that moment on, the silica network is clearly reconstructed from the external surface inwards along the radial direction. However, total recovery does not occur and some porosity can be observed at long times (100 ps), i.e., when the track is already cold. Beyond certain radius, in this case *R* ~ 5.2 nm, density variation is not observed, which means that *R* represents the track size. The average radial velocity becomes positive and negative indicating the mass transport from and to the track region. The pressure gradient and the fact that transport along the cold region is hampered results in a net effect dominated by transport to the track region, which produces a considerable density rise in the track volume. Note that the low density core only accounts for a very small fraction of the total track volume from which atoms moved towards the surrounding denser halo. The observed net density increase is due to a net increase in the number of atoms in the track volume. These atoms necessarily reach the track region from the volume around the track. It is obvious that the arrival of a reduced number of atoms to the small volume of the track results in a significant density increment, whereas the loss of these atoms from the surrounding volume (*R* > 5.2 nm) has not any noticeable influence on the density of this large region.

**Figure 5:**
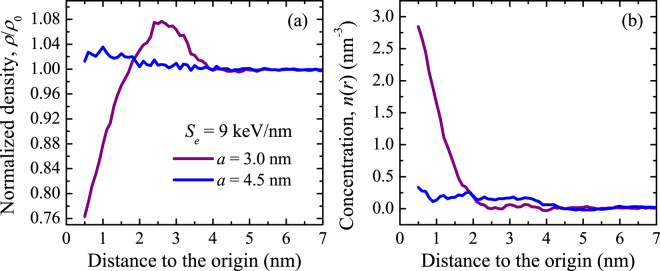
Normalized density (**a**) and colour centre concentration (**b**) obtained by MD simulations with different hot cylinder radii (3.0 and 4.5 nm) as a function of the distance to the track centre for an electronic stopping power of 9 keV/nm. Every point in the figures is obtained by considering all the atoms at the indicated radial distance from the track centre ±0.5 nm.

As shown in Fig. 4 for the example with *S*_e_ = 16 keV/nm, MD simulations provide detailed information about the resulting track structure. Another example is given in Fig. 5(a), which shows the atomic density of cold tracks (100 ps) obtained with different initial hot cylinder radii for *S*_e_ = 9 keV/nm. As in the previous example, the average track density increases with respect to the density of the virgin material. However, the complex core-halo structure is only evident for *a* = 3.0 nm and not for *a* = 4.5 nm. In all our simulations, for a given stopping power, the most pronounced tracks are obtained, as expected, for the smallest hot cylinder radii, (i.e., maximum deposited energy densities).

In addition to determining temperature, pressure and density in the track region, the MD simulations turn out appropriate to study colour centre generation by means of bond breaking followed by incomplete local atomic re-arrangement (see Methods). The total colour centre concentration is obtained in our simulations by adding up the concentrations of all the colour centres. This allowed us to compare experiments and simulations as discussed in the next section. As an example for *S*_e_ = 9 keV/nm, Fig. 5(b) shows the total colour centre concentration obtained with different initial hot cylinder radii in cold (100 ps) tracks. As expected, the colour centre concentration increases with decreasing *a*. The colour centres are contained within a region where temperature reached a high enough value to produce bond breaking that could not be restored during the subsequent fast cooling, whereas the density variation affects a different region along which atomic transport was possible due to the large temperature and pressure gradients that were established for sufficiently long times. In both cases, the permanent effects are the result of the sudden lattice energy rise followed by the ultra-fast cooling that impedes complete network reconstruction.

## Discussion

In the previous section, we showed results on track formation and accumulation that lead to density changes and colour centre generation and in both cases literature data were added for comparison. In addition, we described our atomistic model for track simulation and showed some illustrative results. In this section we will use the experimental results for validation of the model and, simultaneously, we will discuss the physical mechanisms of track formation with the aid of the model.

### Velocity and thresholding effects

Figure 2(a) shows the track cross sections and Fig. 2(b) the track radii obtained by MD with different hot cylinders (indicated by colour code) as a function of the stopping power. The experimental values obtained with different specific energies are plotted together in the figures. According to the simulations, *σ* increases linearly for sufficiently low *S*_e_. In the case of *a* = 3.0 nm, the behaviour tends to become sublinear for *S*_e_ > 10 keV/nm. The experimental data seem to confirm this, although not conclusively due to the scarcity of data points. For high stopping powers the results tend to approach a universal behaviour. Comparing the slopes obtained by a linear fit to the experimental and MD cross sections we can relate the hot cylinder radii to specific energy ranges. For example, the effects produced by ions in the range < 1 MeV/u are fairly well reproduced with *a* = 3.0 nm, whereas the specific energies ~5 MeV/u are reproduced with *a* = 4.5 nm. This provides a *calibration method* for our MD simulations, essential to obtain quantitative results that can reproduce the experiments with just one free parameter: *a*. Without such a calibration, our simple method to simulate energy transfer to the ions would be merely qualitative. The link between the specific energies and the hot cylinder radii provides a meaningful interpretation of the hot cylinders, i.e., they are related to the ion specific energies through the deposited energy density. For a given *S*_e_ value, the deposited energy density decreases quadratically with increasing *a* and therefore, tracks become less pronounced as the energy density decreases. Experimentally, the ion specific energy leads to the same effect. This is due to the fact that the energy transferred from the projectile ion to electrons increases with the ion energy. Thus, the resulting ballistic electrons deposit their energy thorough ionizations and excitations into a volume that increases with the electron energy. This is the origin of the velocity effect^40–44^. Previous MD simulations^29, 32^ did not study in detail the effect of the initial deposited energy density and therefore, did not provide an interpretation of the velocity effect.

Related to the velocity effect there exists a thresholding effect, i.e., sufficiently high stopping powers are needed for track formation. The extrapolation of the track cross section in the linear regime to *σ* = 0 (Fig. 2) provides the threshold stopping powers: *S*_th_ = 1.4 keV/nm for *a* = 3.0 nm and *S*_th_ = 2.5 keV/nm for *a* = 4.5 nm. The agreement with the experimental values is very good for the studied specific energy ranges. From these thresholds, we can estimate the threshold energy density for track formation, which turns out to be 0.6–1.0 eV/atom. Therefore, this is actually the value that must be reached to generate a permanent track. Obviously, if the hot cylinder area increases, it is necessary to increase the stopping power to reach this density level. Similarly, the stopping power required to reach this threshold in the experiments increases for increasing specific energy as a consequence of the velocity effect.

### Track structure

The MD simulations capture the basic features of the generated tracks, i.e., the overall density increase with a low density region in the centre, as shown in Figs 4 and 5. Large hot cylinders reproduce high specific energy ranges, resulting in less marked tracks than in the case of small hot cylinders, to the extent that the core may not appear, as in the example shown in Fig. 5(a) for *a* = 4.5 nm. The linear fit of the core cross sections obtained by MD with *a* = 3.0 nm provides the same slope as the linear fit to the experimental values (Fig. 2), but the MD values are systematically higher than the experimental ones. Long term diffusive effects are not taken into account in the MD simulations. Such effects most likely take place in the experiments, provided the large density gradients obtained at 100 ps (cold tracks). This may explain why the MD core cross sections (core radii) turn out higher than the experimental ones. The fact that the slopes coincide indicates that the physical processes simulated by MD are likely to be the dominant ones in track formation. We can estimate in this case the threshold energy density for core formation considering the experimental stopping power threshold, 2.7 keV/nm and *a* = 3 nm, which results in 1.5 eV/atom, high enough to produce massive bond breaking, according to our simulations. This value is considerably higher than the threshold for track formation estimated in the previous section (0.6–1.0 eV/atom).

Initially, high energy atoms from the central region move radially outwards, leading to a markedly low density region in the centre. It is interesting to note that the average track density increases as a consequence of atom arrival from the surroundings. The net collective atomic motion to the track is favoured over atomic motion from the track region to the surroundings due to the enhanced diffusion in the hot region, whereas atomic motion is hampered in the cold medium outside the track region. In addition, the high compressive pressure (established for tens of ps in the example of Fig. 4) drives atoms to the central region. This pressure in the track is not high enough to lead to phase transitions in silica, according to studies using the same interatomic potential^57^. The process of track formation is initially governed by atomic motion. The subsequent relaxation depends on other parameters such as the pressure gradient and the cooling rate, which ultimately determine the fine details of the permanent track. For example, a permanent low density core only appears when the initial collective atomic motion is so efficient that the number of displaced atoms towards the track edge cannot be compensated by the subsequent arrival of atoms from outside the track region. We can roughly attribute the core formation to high temperature, i.e., atoms gain enough kinetic energy to efficiently move far from their original positions. On the other hand, the high density halo region is mostly caused by a net atomic motion into the track as a consequence of the large temperature and pressure gradients. The resulting tracks are “frozen” in an intermediate metastable state between the initial situation (relaxed atomic network) and the situation of maximum disorder in the network as a result of the fast cooling that suppresses full relaxation.

### Colour centre generation

MD simulations^31^ were successfully used to study the cooperative effect of electronic and nuclear stopping regimes on defect generation by Au ion irradiation. Contrary to that study, we concentrated only on the electronic stopping regime and for this reason, we selected medium mass ions (F, Br and Fe) with a low nuclear stopping power. In addition, we validated the defect generation predicted by our MD simulations with (direct) optical absorption measurements of defect generation, as shown next.

**Figure 6:**
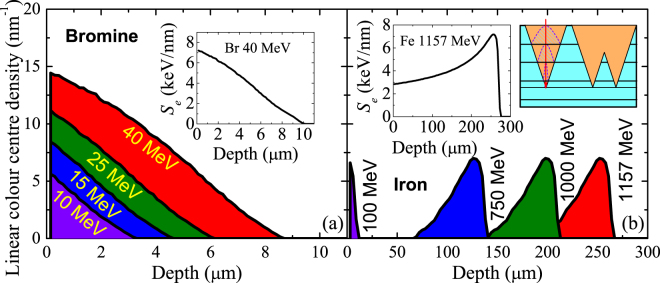
Linear colour centre density, *N*(*z*), obtained by MD for Br irradiation (**a**) and Fe irradiation (**b**) at the indicated ion energies. The shaded areas correspond to the colour centre yield per ion in every irradiation case. The insets contain the electronic stopping powers as calculated by SRIM for two cases. The scheme illustrates the accumulation of tracks and how this process is simulated with a home-made Monte Carlo code that makes use of the colour centre concentrations obtained by MD at different depths, corresponding to different stopping powers (see Methods).

From our MD simulations we can calculate the linear colour centre density (Fig. 6) for the Br and Fe irradiations, ion energies 10–40 MeV and 1157 MeV, respectively (more information in Methods). Simple integration provides the colour centre yields, which are plotted in Fig. 3(a) together with the experimental results. The agreement is remarkable, especially considering the wide range of ion energies involved. The colour centre yields increase with ion energy and as expected, ions of low specific energy (Br series) are more efficient producing colour centres than ions of high specific energy (1157-MeV Fe). At sufficiently high ion energy the simulations predict a maximum yield. This is easy to understand with the aid of Fig. 6. The electronic stopping power for high energy ions is very small, therefore, they must loose energy prior to being effective for colour centre production. In this situation, increasing the ion energy does increase the colour centre mean range but does not increase the colour centre yield. For example, 1157-MeV Fe ions lead to the same colour centre yield as 750-MeV Fe ions.

From MD results, like those in the example of Fig. 5(b), we can estimate the colour centre concentration, *n*(*r*), around the ion track for a given stopping power. Using several MD simulations, we can reproduce the whole ion track and obtain the colour centre concentration as a function of depth, *n*(*r,z*), for every incident ion energy. Then, with the aid of a Monte Carlo computational strategy^58^, we can follow the random ion arrival to the sample surface and the resulting colour centre accumulation as a function of fluence (schematically represented in the inset of Fig. 6, more details in Methods). In this way, we obtained the maximum colour centre areal density for every ion. The quantitative agreement between our atomistic model and experimental data is very good, see Fig. 3(b). Not only is the trend correctly estimated, but also the quantitative values over a large range of ion energies.

**Figure 7:**
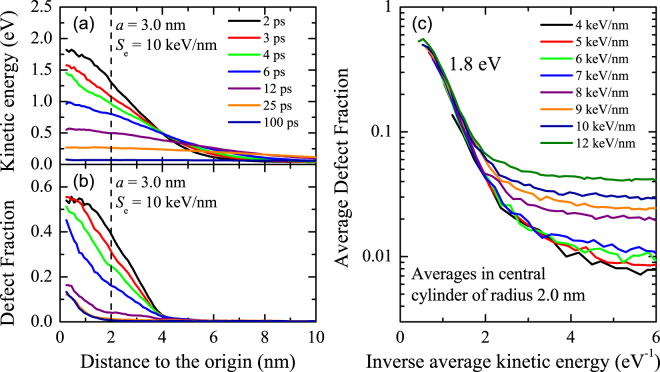
Kinetic energy (**a**) and defect fraction (**b**) as a function of the distance to the track centre for different times after ion arrival as obtained from MD simulations with an initial hot cylinder radius *a* = 3.0 nm and electronic stopping power *S*_e_ = 10 keV/nm. Every point is obtained by considering all the atoms at the indicated radial distance from the track centre ±0.25 nm. The vertical lines indicate the central region (2 nm radius) used to get the average defect fraction, plotted in (**c**) as a function of the inverse average kinetic energy for different stopping powers. When the track remains hot, defect generation follows an Arrhenius behaviour with a characteristic energy of 1.8 eV for all the irradiations, regardless the stopping power.

So far in this section, we have focused on the permanent defects (colour centres) generated by high electronic excitation mechanisms, i.e., those that remain in the track once thermalized. In order to understand the physical mechanisms of defect generation, we need to consider the temporal defect evolution. The sudden lattice energy rise brings the system out of equilibrium, however local equilibrium is rapidly restored within a few ps. Figure 7(a) shows the kinetic energy (temperature) as a function of the distance to the track centre for different times after the ion arrival, obtained from the MD simulations carried out with *a* = 3.0 nm and *S*_e_ = 10 keV/nm. Gaussian-like profiles are already observed in the first picoseconds and then they broaden as a function of time, due to the thermal dissipation. The defect fraction profiles obtained for the same times are plotted in Fig. 7(b). Obviously, they are related to the kinetic energy profiles. In fact, the maximum defect fraction coincides with the maximum temperatures; i.e., it is reached in the central region for short times. Then, the defect fraction swiftly drops as temperature decreases, due to atom rearrangement. Clearly, perfect rearrangement does not occur in the track centre, where the maximum defect fraction was reached, due to the low atomic density of this region (core). Fast cooling suppresses full network reconstruction and leads to a profile of permanent defects, which is the origin of the colour centres observed experimentally. These observations can be extended to ions with different stopping powers. For comparison, we considered a small central region (2-nm radius), where most defects appear. In Fig. 7(c), we plot the average defect fraction in the central region as a function of the inverse average kinetic energy (average local temperature). Remarkably, the average defect fraction in the region where the track remains hot does not depend on the stopping power but only on the temperature. For hot tracks, the average defect fraction follows an Arrhenius behaviour with a characteristic energy of 1.8 eV. This behaviour indicates that local balance between defect formation and defect annealing is achieved for high temperatures. As the temperature drops, such a balance is not sustained anymore because defect annealing becomes slow and is eventually *de facto* suppressed. The result is clearly seen in Fig. 7(c): a permanent defect fraction remains in the central region of cold tracks. The higher the stopping power, the higher the permanent defect fraction that remains. The model indicates that the permanent defects are a consequence of bond breaking and the incomplete thermal annealing of defects as a result of the fast cooling. Pressure-induced defect formation is not observed because the achieved pressures are too low and sustained for a period too short to generate defects, unlike in the presence of stronger steady shock waves^57^.

### Track models

The atomistic model described in this paper is able to quantitatively reproduce a broad range of ion irradiation-induced high electronic excitation experiments in silica. This includes compaction, leading to dielectric constant variation and defect generation with ions of very different specific energies (velocity effect). The model provides a detailed description of the track region evolution after the sudden energy deposition, which triggers the processes that lead to track formation. Phenomenological models able to provide a good estimate of the temperature reached in the track are candidates to make good predictions concerning experimental track size with just a few assumptions. Indeed, thermal models, such as the two-temperature model^9^, can describe track formation in silica whereas other phenomenological descriptions cannot. However, understanding damage creation requires both disordering and recombination contributions. Failure to consider one of them in a model can lead to considerable inaccuracies in the predictions. The phenomenological models mentioned in the Introduction section^9, 14–20^ can hardly consider these two terms, whereas, the atomistic modelling presented here combines them naturally. For example, the complex track structure (low density core and high density halo) observed experimentally^32^ does not depend only on the maximum temperature reached. In our model, it is a consequence of a sudden rise in kinetic energy leading to collective atomic motion from and to the track region, pressure-driven atom diffusion and ultra-fast cooling resulting in incomplete network recovery. The final configuration of permanent tracks is obviously dominated by the initial energy deposition but also depends on the details of network reconstruction during the cooling stage. Another example, which turns out hard to model, is colour centre generation. As shown in this paper, the final defect configuration is a consequence of massive bond rupture and incomplete thermal annealing. While the temperature is high, local balance between generation and annealing occurs. However, the equilibrium does not hold anymore as the temperature drops because annealing mechanisms become too slow. The result is that a significant fraction of the generated defects remains once the track is cold. This fraction strongly depends on the fine details of bond reconstruction, which obviously depend on temperature but also on the atomic density in the track.

The fact that our MD simulations are compatible with many aspects of complex track formation indicates that the method can be applied to this and other irradiation cases and it is expected that it will provide acceptable quantitative predictions. An open question, not addressed in this paper, is related to the experimentally observed defect generation in subthreshold irradiations at high fluence^59^; i.e., in the track overlapping regime.

## Methods

### Experimental

We carried out irradiation experiments at Centro de Micro-Análisis de Materiales (CMAM-UAM) with the 5 MV tandem accelerator. Ions (Br at 5, 10, 15, 25 and 40 MeV and F at 5 MeV), i.e., in the electronic stopping regime (2–8 keV/nm) were implanted into low OH content (below 10 ppm) silica samples provided by Momentive Ltd. The ion currents were kept below 30 nA in every case to avoid sample overheating.

An optical reflection setup was mounted in the irradiation chamber to carry out *in situ* measurements, as we have described elsewhere^38^. Measurements were taken in nearly-normal conditions with the aid of two mirrors. A halogen lamp was used for illumination. The reflected component was focused with a 4-cm focal length lens onto a silica optical fibre (1-mm diameter) to guide the reflected light to a compact spectrometer (QE6500 Ocean Optics Inc.) configured with a multichannel array detector able to measure the whole spectrum from 200 to 1000 nm with a spectral resolution better than 2 nm. The integration time during the measurements was 1 s for the range 500–900 nm. The chosen integration time assures that variations in the dielectric constant as a function of fluence can be followed with great detail, as seen in Fig. 1.

In addition, *ex situ* optical absorption measurements in VIS-UV range up to 8.2 eV were carried out in transmission configuration to detect the most abundant colour centres^39^. A number of samples (6 to 8) were irradiated at different fluences for every ion energy. The deconvolution of the absorption spectra to obtain the colour centre concentrations over the whole sample depth was carried out with the parameters listed in Table 2.

### Atomistic model

In order to model the thermal evolution of the track region in silica, we performed atomistic simulations based on molecular dynamics (MD) using the Feuston-Garofalini interatomic potential^60^, modified by the Ziegler, Biersack and Littmark (ZBL) potential to account for close interactions. This potential has been successfully used to describe shocks in silica under extreme conditions^57, 61^. Simulation boxes of 30 × 30 × 14 nm^3^ of β-cristobalite, first melted and then relaxed at 300 K were prepared as described elsewhere^30^. Periodic boundary conditions were applied to all directions. The total number of atoms in the box was ~8.5 × 10^5^ and the simulation times exceeded 100 ps. The employed code was MDCASK run in 256−512 cores at supercomputer CESVIMA-MAGERIT (UPM).

In our MD simulations, the energy transferred by the ion is introduced as kinetic energy of the atoms in a (hot) cylinder of radius 3–5 nm^21–27^. A sufficiently large concentric cylinder (radius 14.3 nm) is used to account for the thermal evolution. The remaining surrounding atoms constitute an appropriate thermal bath to describe the radial heat transfer outwards the hot cylinder. The thermal bath temperature is reset to 300 K every 10 steps, i.e., 5 fs, by rescaling the velocity of the atoms in the bath. Analogously to an ensemble of harmonic oscillators, the kinetic energy equilibrates with the potential energy in a fraction of a ps and, subsequently, heat transfer to the surrounding bath leads to a Gaussian-like temperature profile, as expected.

The box dimensions were chosen as a good compromise to minimise the statistical fluctuations at reasonable computational times. Every simulation lasted 10–20 h, an acceptable time for this simulation campaign that involved more than 100 simulations. The statistics are in general acceptable and allowed us to extract quantitative information from the simulations. The most difficult analysis was that of the track core (radius 1–2 nm) due to the small number of atoms in such a small volume. In order to smooth in a natural way the curves corresponding to different parameters obtained in cylindrical coordinates as a function of distance to the track centre (density, colour centre concentration, velocities, pressure), we calculated every parameter as a function of the distance ± 0.25 or 0.5 nm. This procedure eliminates artefacts stemming from statistical fluctuations at the expense of reducing the radial resolution.

Experimentally, we detect optically active defects (colour centres) identified as E’-centres, non-bridging oxygen hole centres (NBOHC) and oxygen deficient centres (ODC). They appear as a consequence of a bond rearrangement process that necessarily involves bond breaking. In our simulations, at sufficiently high stopping powers, massive bond breaking followed by rapid atom rearrangement is observed. In order to quantify colour centre production, we count the number of Si atoms with coordination number 3 (precursors for E’-centres and ODC) and O atoms with coordination number 1 (precursors for NBOHC), assuming that the Si-O bond length is < 0.2 nm. When the number of under-coordinated atoms produced is very high (that is, when massive bond breaking takes place) the initial lattice is not fully restored due to the fast cooling.

The optical absorption measurements presented in Fig. 3 are the result of all the colour centres generated along the ion range in the experiments, where the stopping power is decreasing as the ion travels through the lattice. Therefore, in order to make a proper comparison with the experimental data, a number of MD simulations with different stopping powers and hot cylinder radii must be performed to represent the experimental effects produced by one single ion of certain energy. This is schematically shown in the inset of Fig. 6. Following the previously discussed link between hot cylinder radius and ion specific energy we took *a* = 3.0 nm to simulate the Br irradiations and *a* = 4.5 nm to simulate the Fe irradiations, corresponding to ions of *E*_s_ < 1 MeV/u and *E*_s_ ~ 5 MeV/u along most of their trajectories, respectively. Combining the simulations for different stopping powers we determine the colour centre yield for Br and Fe irradiations as follows. Each simulation provides the colour centre concentration profile around the ion trajectory, *n*(*r*), see e.g., Fig. 5(b). By cylindrical integration we obtain the linear colour centre density *N* for the simulated stopping power. Combining the simulations at different stopping powers, we obtain a function *N*(*S*_e_) by linear interpolation (obviously, a number of simulations at different stopping powers covering the whole ion range are needed for this purpose). SRIM provides the *S*_e_ dependence with depth, *S*_e_(*z*) (insets in Fig. 6) and we use this relation to convert *N*(*S*_e_) to the linear colour centre density as a function of depth, *N*(*z*). Thus, we can obtain the colour centre yield per ion, simply by integrating *N*(*z*) over the depth, i.e., the shaded areas under the curves in Fig. 6 correspond to the colour centre yield per Br ion (a) and per Fe ion (b) at different ion energies. We used this procedure to obtain the colour centre yields shown in Fig. 3(a).

As shown in the Results section, colour centre production as a function of ion fluence follows a Poisson-like behaviour, indicating that colour centre generation by an ion impact is in first approach independent of the existing colour centre concentration. In order to simulate this process, we need to take into account the random arrival of ions to the sample surface. For this purpose, we developed a Monte Carlo code analogous to the one that we used in a previous work^58^. We considered a box with a surface area of 100 × 100 nm^2^ and a depth similar to the ion range. We divided the box in typically 5 slabs of cells. Their thickness is chosen in such a way that the stopping power does not change significantly along it. The cells have surface dimensions of (1 × 1) nm^2^. For a given ion energy, we can calculate by means of SRIM the ion stopping power as a function of depth. Using several MD simulations at different stopping powers (one per slab), we obtain a reasonably accurate colour centre concentration profile for every slab (depth). Then, the impact point coordinates (*x*, *y*) are determined randomly. Therefore, upon the arrival (normal to the surface) of every ion we included the generated colour centre concentrations in the Monte Carlo box by modifying the cells around the (*x*, *y*) cell in every slab according to the obtained MD concentration profiles for that slab (depth, i.e., stopping power). This process is schematically represented in the inset of Fig. 6(b). An isolated track is considered in the Monte Carlo code by assigning the right values of colour centre concentration to the cells around the ion trajectory for every slab (depth). Track overlapping arises naturally when cells previously containing a certain colour centre concentration (due to a previous ion impact) are modified as a result of a new ion impact in their vicinity. Obviously, for very high ion fluences, all the cells in the ion range are affected sooner or later by the passage of one or more ions. Then, colour centre saturation occurs because the net colour centre concentration cannot be modified anymore. Note that the optical absorption measurements detect all the colour centres along the ion range. For this reason, in order to compare with experiments, we ran the Monte Carlo code until saturation was reached and calculated the total areal density for every ion energy. The resulting saturation areal densities for several ions are shown in Fig. 3(b) and compared with experimental values.

## Conclusions

In this paper, we have studied the effect of ion irradiation-induced high electronic excitation in silica, by means of experiments and simulations. The experiments provided information on the resulting track radius, dielectric constant variation (and thus, compaction) and defect formation. The simulations were based on an atomistic model to study the evolution of the material upon ion irradiation. The model was applied to the study of different ion irradiation experiments in which energy deposited into the electronic system eventually heats up the lattice in the track region. The model is validated by our experiments combined with results from the literature. The obtained results indicate that the sudden lattice energy rise in the track region by the incoming ions leads to a massive disorder of the material and bond breaking. The atoms in the central region get very high kinetic energy. The result is a collective motion radially outwards from this region. Subsonic mass transport takes place in the track region. The net effect is mass transport from the surrounding volume into the track as a consequence of the induced high temperature and pressure gradients. A metastable permanent track with an average density higher than the virgin material is formed after ultra-fast cooling. We obtained a threshold energy density for track formation around 0.6–1.0 eV/atom. The tracks generally have a complex structure, with a low density core and a high density halo. The threshold for core formation is 1.5 eV/atom. Therefore, the core corresponds to the region where the highest temperatures were reached and the halo is formed by the accumulation of atoms from the core and from the surrounding cold region. These thresholds are also related to the velocity effect, i.e., the higher the ion specific energy, the more difficult to reach these threshold densities. Defects are generated in the track and the permanent defect fraction is a consequence of the unbalance between defect formation and recombination as temperature swiftly drops. The behaviour in the track centre follows an Arrhenius law with a characteristic energy of 1.8 eV. The experimental observations are compatible with the evolution of the track region after the sudden energy deposition by the ion provided by the model. The intricate physical mechanisms taking place lead to permanent effects difficult to predict quantitatively by means of simple models. Based on the results of this paper, the application of the methods to other materials and irradiation conditions seems feasible. This opens possibilities for improved nanostructuring methods based on ion irradiation, of technological interest in several fields.
